# Clinical Impact of Cytomegalovirus Infection in Patients with Inflammatory Bowel Disease

**DOI:** 10.5152/tjg.2026.25407

**Published:** 2026-01-08

**Authors:** Bengü Dursun, Mesut Gumussoy, Murat Törüner

**Affiliations:** 1 Department of Internal Medicine, Ankara University Faculty of Medicine, Ankara, Türkiye; 2 Department of Gastroenterology, Ankara University Faculty of Medicine, Ankara, Türkiye

**Keywords:** Cytomegalovirus, CMV colitis, CMV-DNA, colectomy, inflammatory bowel disease, prognosis

## Abstract

**Background/Aims:**

: Patients with inflammatory bowel disease (IBD) are at increased risk of cytomegalovirus (CMV) infection, likely due to malnutrition and immunosuppressive therapies. Cytomegalovirus infection may contribute to worse disease outcomes, including higher colectomy rates. However, the prognostic value of serum cytomegalovirus deoxyribonucleic acid (CMV-DNA) levels and the clinical significance of CMV colitis remain unclear. This study aimed to assess the impact of CMV infection on the clinical course of IBD.

**Materials and Methods:**

: Retrospective analysis was performed on 141 hospitalized IBD patients with detectable serum CMV-DNA (>42 copies/mL). Cytomegalovirus colitis was diagnosed by histopathology or immunohistochemistry. Clinical features were compared between CMV colitis and non-colitis groups. Receiver operating characteristic analysis assessed the optimal CMV-DNA cut-off, and logistic regression identified predictors of adverse outcomes (mortality or colectomy).

**Results:**

: Cytomegalovirus colitis was identified in 18.4% of patients. While there were no significant differences in age, treatment history, or colectomy rates between groups, patients with CMV colitis had significantly higher serum CMV-DNA levels (median: 837 vs. 267 copies/mL; *P* = .019), longer hospitalization durations (*P* = .001), and more frequent comorbidities (*P* = .019). The optimal CMV-DNA cut-off was 468.5 copies/mL (area under the curve (AUC) 0.64; sensitivity 61.5%, specificity 61.7%). Adverse outcomes occurred in 24% of cases. Extensive colitis (odds ratio 2.92; *P* = .034) independently predicted poor outcomes; CMV-DNA levels did not.

**Conclusion:**

: Cytomegalovirus colitis is an uncommon but clinically significant complication in IBD flares. Although high serum CMV-DNA levels were associated with CMV colitis, their diagnostic value was limited. Extensive colitis is an independent predictor of poor outcomes.

**Main Points**

- Serum cytomegalovirus deoxyribonucleic acid (CMV-DNA) levels were significantly higher in patients with histologically confirmed CMV colitis, but its diagnostic accuracy was limited.
- Cytomegalovirus colitis was associated with longer hospital stays and higher prevalence of comorbidities in inflammatory bowel disease (IBD) patients.
- Extensive colitis independently predicted adverse outcomes such as colectomy or in-hospital mortality.
- Serum CMV-DNA polymerase chain reaction (PCR) should not be used as a standalone diagnostic tool for CMV colitis.
- Cytomegalovirus reactivation may reflect underlying disease severity rather than being a direct cause of adverse outcomes.

## Introduction

Inflammatory bowel diseases (IBD), encompassing ulcerative colitis (UC) and Crohn’s disease (CD), are chronic immune-mediated disorders of the gastrointestinal tract with a relapsing-remitting clinical course.^1^^,^^2^ Despite advances in therapeutic strategies, a significant subset of patients experiences acute exacerbations that might require hospitalization, high-dose corticosteroids, and escalation to immunosuppressive or biologic therapies. In this clinical setting, the risk of opportunistic infections becomes a significant concern.^3^

Cytomegalovirus (CMV) is a member of the β-herpesvirus family that remains latent after primary infection, with reactivation occurring in immunocompromised hosts.^4^ Exposure to corticosteroids or thiopurines has been associated with CMV reactivation in patients with IBD, especially during severe disease flares.^5^ Although CMV colitis is a well-documented clinical entity, its main role in worsening the disease course of IBD remains controversial. The major clinical dilemma is whether CMV acts as a true pathogen that exacerbates mucosal inflammation or merely as a bystander secondary to severe colonic inflammation.^6^

Although numerous diagnostic modalities—such as serology, histopathology, or tissue polymerase chain reaction (PCR)—are available for detecting intestinal CMV infection, there is no clear consensus in the literature on the most accurate method.^7^ However, in the most recent European Crohn’s and Colitis Organisation guidelines it has been stated that immunohistochemistry (IHC), possibly tissue PCR, or both, are essential for confirming active CMV colitis in IBD and should be the standard tests (*Journal of Crohn’s and Colitis*, 2021, 879-913). In recent years, noninvasive methods such as quantitative PCR assays of serum or plasma cytomegalovirus deoxyribonucleic acid (CMV-DNA) have emerged as valuable tools for detecting systemic CMV viremia.^8^ The ability of serum CMV-DNA positivity to predict adverse clinical outcomes in IBD, including steroid refractoriness, prolonged hospital stay, colectomy, or mortality, remains uncertain in current literature.

Given the clinical ambiguity surrounding CMV reactivation in IBD, there is a growing need for better understanding of its impact on disease outcomes. Establishing whether serum CMV-DNA PCR can serve as a reliable marker for adverse prognosis may help guide therapeutic decisions, including the need for antiviral treatment and escalation of IBD-directed therapy. This retrospective study evaluated the clinical significance of CMV infection, as detected by serum CMV-DNA PCR, in patients admitted for moderate-to-severe IBD exacerbations.

## Materials and Methods

### Study Cohort

This retrospective single-center study included patients admitted for IBD (UC and CD) flare-ups with detectable serum CMV-DNA levels (>42 copies/mL) at the Department of Gastroenterology, Ankara University School of Medicine (Ankara, Türkiye) between 2006 and 2016. The IBD flare-up was defined as a clinical worsening requiring hospitalization and either re-initiation of corticosteroid or escalation to new therapeutic agents. As part of the standard diagnostic work-up performed during the flare episodes, stool microscopy, stool culture, Clostridium difficile toxin A/B assays, and serum CMV-PCR were routinely performed. Colonoscopy was performed in all patients to determine the extent of bowel involvement and to assess disease activity. Extensive colitis was defined as disease extending proximal to the splenic flexure and involving the cecum. Relevant demographic, clinical, and laboratory variables were collected from the patients’ electronic medical records. This study was approved by the Clinical Research Ethics Committee of Ankara University School of Medicine on December 12, 2016 (19-960-16). Informed consent was not obtained due to the retrospective design of the study.

### Diagnosis of Cytomegalovirus Colitis and Clinical Outcomes

During endoscopic evaluation for an IBD flare, CMV colitis was diagnosed if at least 1 inclusion body was detected by hematoxylin and eosin (H&E) or IHC staining in the colorectal mucosa obtained by endoscopic biopsy. In this study, adverse clinical outcomes were defined as colectomy or death resulting from the current IBD exacerbation. The impact of serum CMV-DNA levels on the presence of CMV colitis and adverse clinical outcomes was evaluated. In addition, clinical and laboratory parameters potentially associated with adverse outcomes were also evaluated.

### Statistical Analysis

Descriptive statistics were presented as the median for continuous variables and percentages for categorical variables. Comparisons between patients with and without CMV colitis were performed using the Mann–Whitney *U*-test for continuous variables and the Chi-square test for categorical variables. The cut-off value for serum CMV-DNA was determined with the receiver operating characteristic (ROC) analysis using Youden’s J index. The impact of high serum CMV-DNA levels, determined based on the established cutoff, on the presence of CMV colitis was evaluated using binary logistic regression analysis. Univariate analyses were conducted to assess associations between clinical variables and adverse clinical outcomes (defined as in-hospital mortality or colectomy). Variables with *P* < .1 in univariate analysis were included in the multivariate logistic regression model to identify independent predictors. Odds ratios (OR) with 95% CI were reported. The statistical analyses were performed with SPSS, version 25.0 (IBM SPSS Corp.; Armonk, NY, USA). A type-I error level of 5% (*P* < .05) was considered the threshold limit for statistical significance.

## Results

### Baseline Characteristics

A total of 1154 patients with IBD were retrospectively evaluated. Among them, 141 patients with IBD flare-ups with detectable serum CMV-DNA levels (>42 copies/mL) were enrolled in the study. Table 1 summarizes the baseline demographic and clinical characteristics of the patients. Most of the patients had a diagnosis of UC (86.5%). The median age was 42.5 years and 35% were female. Of these patients, 26 (18.4%) were diagnosed with CMV colitis. Of the 26 patients diagnosed with CMV colitis, IHC of colonic biopsies was positive in 24 cases, whereas CMV inclusion bodies were identified by H&E staining in 18 cases; both diagnostic modalities were concurrently positive in 16 patients. Antiviral therapy was administered to 80.7% of patients with CMV colitis compared with 33% of those with CMV-DNA PCR positivity (*P* = .001). Patients with CMV colitis had significantly longer hospital stays and a higher prevalence of comorbidities (*P* = .0001 and .019, respectively). However, there was no statistically significant difference in age, disease extent, IBD treatment, laboratory parameters, or colectomy rates between patients with and without CMV colitis (Table 2).

### The Association Between Serum Cytomegalovirus-DNA Levels and Cytomegalovirus Colitis

**Figure 1.**
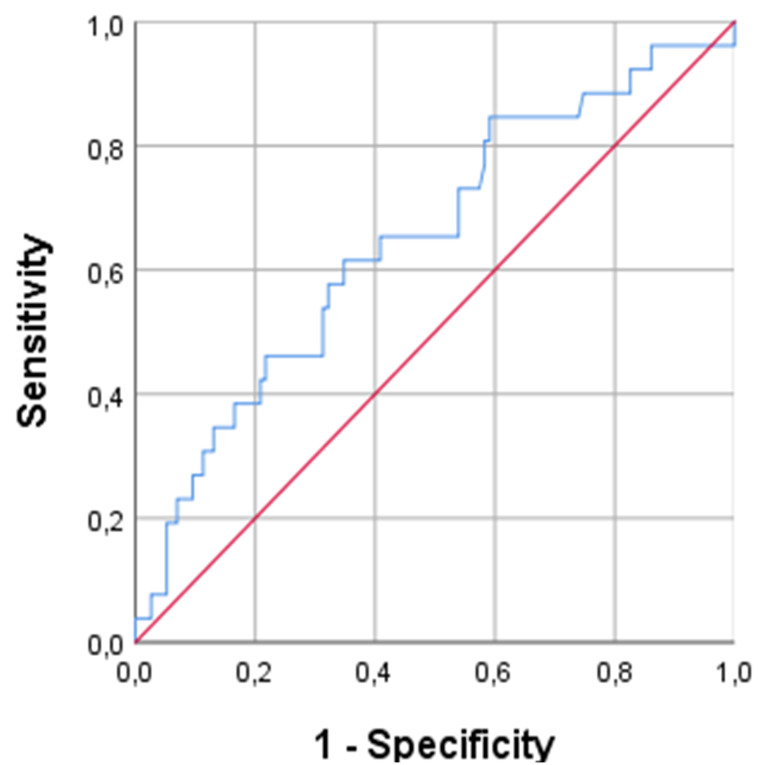
Receiver operating characteristic analysis of serum CMV-PCR for predicting CMV colitis.

In this cohort, the overall initial median serum CMV-DNA PCR level was 316 copies/mL. Receiver operating characteristic curve analysis identified 468.5 copies/mL as the optimal cutoff value for predicting CMV colitis, with a sensitivity of 61.5% and specificity of 61.7% (area under the curve (AUC): 0.64; 95% CI: 0.52-0.76; *P* = .019) (Figure 1). When stratified by serum CMV-DNA PCR levels using a cutoff of 468.5 copies/mL, patients with higher viremia exhibited significantly prolonged hospitalization (median 23 vs. 8 days, *P* = .001). No statistically significant differences were observed between groups in terms of age, disease extent, CRP levels, hemoglobin, colectomy rates, or exposure to immunosuppressive agents (Table 3).

### Adverse Clinical Outcomes

Adverse clinical outcomes were observed in 24% of the cohort. Colectomy or mortality occurred more frequently among patients with extensive colitis, elevated serum CMV-DNA levels and prolonged hospitalization (Table 4). Glucocorticoid dependency (*P* = .075) and anti-TNF exposure (*P* = .094) were related to worse outcomes; however, these effects did not reach statistical significance. In addition, patients who received antiviral therapy seemed to have worse outcomes as well (*P* = .074). Among patients with CMV colitis who received antiviral therapy, adverse clinical outcomes were observed in 8 cases (30%). In the multivariate logistic regression analysis using a binary cut-off for serum CMV-DNA levels, extensive colitis remained an independent risk factor for adverse clinical outcomes (OR: 2.92, 95% CI: 1.08-7.85, *P* = .034). While patients with CMV-DNA PCR levels above 468.5 copies/mL showed a trend toward worse outcomes, this association did not reach statistical significance (OR: 2.04, *P* = .135). Duration of hospitalization was not associated with clinical outcome (*P* = .99) (Table 5).

## Discussion

In this retrospective study, the clinical impact of CMV viremia and colitis in patients hospitalized with moderate-to-severe IBD flares was evaluated. Among 141 patients screened over a 10-year period, CMV colitis based on H&E or IHC staining in the colorectal mucosa obtained by endoscopic biopsy was detected in 18.4% of cases. Similarly, previous studies have reported that the prevalence of CMV infection in acute severe colitis ranges from 21% to 34%, depending on disease severity and the extent of immunosuppressive therapy.^9^^,^^10^ In the cohort, the vast majority of patients (92.3%) were diagnosed with CMV colitis by tissue IHC, which is regarded as the diagnostic gold standard. Notably, the remaining 2 patients demonstrated only inclusion bodies on H&E staining despite negative IHC results. While this discrepancy may reflect potential false-positive diagnoses, it nevertheless remains consistent with previously reported findings in the literature.

The findings revealed that patients with histologically confirmed CMV colitis had significantly higher serum CMV-DNA levels compared to those without colitis (837 vs. 267 copies/mL, *P* = .019). Although serum CMV-PCR is a rapid and noninvasive tool, its diagnostic performance was poor in the study, with ROC analysis identifying 468.5 copies/mL as the optimal cut-off sensitivity 61.5%, specificity 61.7%, AUC = 0.64. A recent meta-analysis evaluating the diagnostic performance of non-invasive serum and stool-based tests for CMV ileo-colitis, serum CMV PCR demonstrated a pooled sensitivity of 62% and specificity of 90%, while stool CMV PCR showed similar specificity 91% but lower sensitivity 53%.^11^ These results highlight the limitations of serum-based diagnostics in detecting mucosal involvement and support the current consensus that serum CMV-DNA PCR should not be used as a standalone diagnostic tool for CMV colitis, but rather as a complementary test to guide further investigation, particularly when tissue biopsies are inconclusive or unavailable.^12^

Although the rates of extensive colitis, steroid dependence, anti-TNF exposure, anti-viral therapy, CRP levels, hemoglobin, leukocyte count, and colectomy rates were not significantly different between patients with and without CMV colitis, there was a tendency toward a more aggressive disease phenotype in the CMV colitis group. Retrospective series suggested that CMV may trigger a steroid refractory flare-up and worsen disease prognosis associated with an increased risk of toxic megacolon and surgical intervention.^13^^,^^14^ Patients with CMV colitis had significantly longer hospital stays (median: 25.5 days vs. 12 days, *P* = .001), suggesting a more severe disease course or delays in clinical response. Prolonged hospitalization may also reflect the complexity of managing these patients, often requiring both immunosuppression and antiviral therapy, as well as multidisciplinary care.^15^^,^^16^ Moreover, comorbidities were significantly more common in the CMV colitis group (*P* = .019), which may have contributed to poorer immune regulation and increased susceptibility to viral reactivation. Finally, the more frequent use of antiviral therapy in patients with CMV colitis may have increased the likelihood of remission, which could partly explain the absence of a difference in adverse clinical outcomes between the 2 groups. Interestingly, the duration of hospitalization, although significantly longer in patients with CMV colitis, was not independently associated with adverse outcome in the multivariate model—possibly due to confounding from disease severity or treatment complexity.

Adverse clinical outcomes—defined as colectomy or in-hospital mortality—were observed in 24% of the cohort. These outcomes were significantly more frequent among patients with extensive colitis, higher serum CMV-DNA levels, and prolonged hospitalization. In multivariate analysis, patients with extensive colonic involvement had nearly a threefold increased risk of adverse outcomes (OR: 2.92; 95% CI: 1.08-7.85; *P* = .034), in line with existing literature showing that extensive disease is a key determinant of prognosis in IBD. Interestingly, duration of hospitalization, although significantly longer in patients with CMV colitis, was not independently associated with adverse outcome in the multivariate model—possibly due to confounding from disease severity or treatment complexity.^15^ The study supports that CMV reactivation in IBD reflects underlying immune dysfunction or mucosal damage rather than acting as a direct pathogen.^6^^,^^17^

This study has certain limitations. It was a retrospective study and the number of patients with confirmed CMV colitis was relatively small, limiting subgroup analyses. The small sample size also reduced the statistical power of multivariate analyses; therefore, the findings should be interpreted with caution. Another limitation of this study is that serum CMV-DNA was assessed only at a single time point, and serial measurements were not available. Dynamic changes in CMV viremia could have provided additional diagnostic and prognostic insights, particularly for distinguishing transient low-level viremia from progressive CMV disease and for monitoring treatment response. Furthermore, this cohort included only patients with detectable serum CMV-DNA at the time of IBD flare; therefore, non-viremic CMV colitis cases were not captured, which may have led to underestimation of the true prevalence and introduced selection bias. Despite these limitations, this study contributes valuable data regarding the epidemiology and prognostic implications of CMV reactivation in IBD, particularly in the setting of disease flares requiring hospitalization.

These findings highlight the importance of considering CMV reactivation as a potential marker of disease severity in hospitalized IBD patients and support the use of serum CMV-DNA levels as a supplementary tool for guiding further tissue-based evaluation and clinical decision-making.

Cytomegalovirus colitis remains a clinically relevant complication during IBD flares, particularly in patients with extensive disease or comorbid conditions. While elevated serum CMV-DNA levels were associated with histologically confirmed CMV colitis and prolonged hospitalization, its diagnostic value is limited, and it should be interpreted in conjunction with clinical and histopathological findings. Extensive colitis remains the most consistent independent predictor of adverse outcomes, underscoring the need for early risk stratification and individualized management strategies in hospitalized IBD patients.

## Figures and Tables

**Table 1.** Baseline Patient Characteristics of Study Cohort

**Baseline Characteristics (n = 141)**	**n (%)**
Age, year (median) (min-max)	42.5 (16-82)
Sex (Male/Female)	92 (65)/49 (35)
Comorbidity (+)	53 (37.6)
UC/CD	122 (86.5)/19 (13.5)
Disease extent of UC Extensive colitis Left-sided colitis Proctosigmoiditis Proctitis	72 (59) 22 (18) 12 (10) 5 (4)
Disease extent of Crohn Ileocolitis Ileitis	15 (79) 4 (21)
Oral 5-ASA agents (+)	138 ( 98.6)
Systemic glucocorticoids (+)	114 (81.4)
Azathioprine (+)	94 (67.1)
Anti-TNF agents (+)	40 (28.8)
Colectomy (+)	21 (14.9)
CRP levels mg/L (median) (min-max)	23.6 (0.4-293)
CMV colitis (+)	26 (18.4)
IHC +	24 (92.3)
H&E staining +	18 (69.2)
IHC and H&E +	16 (61.5)

5-ASA, 5-aminosalicylic acid; Anti-TNF, anti-tumor necrosis factor; H&E, hematoxylin and eosin; IHC, immunohistochemistry; UC, ulcerative colitis.

**Table 2.** Comparisons of Baseline Characteristics in the CMV Colitis and Non-CMV Colitis Group

	**CMV Colitis** **(CMV PCR+/Biopsy+)** **n = 26**	**Non-CMV Colitis** **(CMV PCR+/Biopsy−)** **n = 115**	*P*
Age (median, IQR)	53 (36-63)	42 (29-56)	.62
UC/CD	23/3	99/16	.74
Extensive colitis (+/−)	17/9	70/33	.80
Glucocorticoid-dependent disease (+/−)	20/6	94/20	.51
Anti-TNF agent (+/−)	4/22	36/77	.094
Comorbidity (+/−)	15/11	38/77	.019
CRP mg/L (median)	26.1	22.7	.58
Hb g/dL (median)	10.6	11	.29
Leukocyte count (median)	7150	7610	.95
Duration of hospitalization (day)(median)	25.5	12	.001
Anti-viral therapy (+/−)	21/5	38/77	.001
Colectomy (+/−)	4/22	17/98	.93

CD, Crohn disease; CRP, C-reactive protein; Hb, hemoglobin; IQR, interquartile range; TNF, tumor necrosis factor; UC, ulcerative colitis.

**Table 3.** Comparison of Baseline Characteristics by CMV DNA PCR Cut-Off (468.5 Copies/mL)

	CMV DNA PCR <468.5	CMV DNA PCR ≥468.5	*P*
Age (median, IQR)	37 (28-52)	43 (28-56)	.093
UC/CD	69/11	53/8	.91
Extensive colitis (+/−)	48/26	39/16	.46
Glucocorticoid-dependent disease (+/−)	63/16	51/10	.56
Anti-TNF agent (+/−)	24/55	16/44	.63
Comorbidity (+/−)	28/52	25/36	.46
CRP mg/L (median)	23.5	25.9	.93
Hb g/dL (median)	11	10.9	.95
Leukocyte count (median)	8100	7300	.40
Duration of hospitalization (day) (median)	8	23	.001
Colectomy (+/−)	11/69	10/51	.66

CD, Crohn disease; CRP, C-reactive protein; Hb, hemoglobin; IQR, interquartile range; TNF, tumor necrosis factor; UC, ulcerative colitis.

**Table 4.** Comparison of Baseline Characteristics of Patients with Spontaneous Remission and Poor Clinical Outcomes

	Adverse Clinical Outcome (n = 35)	Remission (n = 99)	*P*
Age (years) (≤40; >40)	15/19	40/57	.77
Extensive colitis (+/−)	28/6	55/35	.025
Glucocorticoid-dependent disease (+/−)	32/3	77/22	.075
Anti-TNF agent (+/−)	4/22	36/77	.094
Comorbidity (+/−)	14/21	37/62	.78
CRP mg/L (<30; ≥ 30)	20/14	55/44	.74
Hb g/dL (<10.5; ≥10.5)	17/18	35/64	.16
Leukocyte count (ULN or not)	8/27	18/81	.54
Serum CMV-DNA PCR level (copies/mL, median)	796	204	.018
CMV colitis (+/−)	7/28	18/81	.81
Anti-viral therapy (+/−)	20/15	38/61	.074
Duration of hospitalization (day, median)	21	15	.022

CRP, C-reactive protein; Hb, hemoglobin; UC, ulcerative colitis; ULN, upper limit of normal.

**Table 5.** Multivariate Logistic Regression for Predicting Adverse Clinical Outcome

Clinical Parameters	Adverse Clinical Outcomes OR (Lower-Upper 95% Cl)	*P*
Extensive colitis (yes/no) Serum CMV-DNA PCR level (>468.5 copies/mL) Duration of hospitalization (>10 days)	2.92 (1.08-7.85) 2.04 (0.79-5.25) 0.99 (0.36-2.73)	.034 .135 .99

CMV-DNA PCR, cytomegalovirus deoxyribonucleic acid polymerase chain reaction; OR, odds ratio.

## Data Availability

The data that support the findings of this study are available on request from the corresponding author.

## References

[1] Baumgart DC Carding SR. Inflammatory bowel disease: cause and immunobiology. Lancet. 2007;369(9573):1627 1640. (doi: 10.1016/S0140-6736(07)60750-8) 17499605

[2] Törüner M Ünal NG. Epigenetics of inflammatory bowel diseases. Turk J Gastroenterol. 2023;34(5):437-448. (doi: 10.5152/tjg.2023.22515) PMC10334590 37158530

[3] Toruner M Loftus EV Jr Harmsen WS Risk factors for opportunistic infections in patients with inflammatory bowel disease. Gastroenterology. 2008;134(4):929 936. (doi: 10.1053/j.gastro.2008.01.012) 18294633

[4] Griffiths P Reeves M. Pathogenesis of human cytomegalovirus in the immunocompromised host. Nat Rev Microbiol. 2021;19(12):759 773. (doi: 10.1038/s41579-021-00582-z) 34168328 PMC8223196

[5] Shukla T Singh S Tandon P McCurdy JD. Corticosteroids and thiopurines, but not tumor necrosis factor antagonists, are associated with cytomegalovirus reactivation in inflammatory bowel disease: a systematic review and meta-analysis. J Clin Gastroenterol. 2017;51(5):394 401. (doi: 10.1097/MCG.0000000000000758) 27875356

[6] Lawlor G Moss AC. Cytomegalovirus in inflammatory bowel disease: pathogen or innocent bystander? Inflam Bowel Dis. 2010;16(9):1620 1627. (doi: 10.1002/ibd.21275) 20232408

[7] Johnson J Affolter K Boynton K Chen X Valentine J Peterson K. CMV disease in IBD: comparison of diagnostic tests and correlation with disease outcome. Inflam Bowel Dis. 2018;24(7):1539 1546. (doi: 10.1093/ibd/izy045) 29718356

[8] Mourad FH Hashash JG Kariyawasam VC Leong RW. Ulcerative colitis and cytomegalovirus infection: from A to Z. Journal. J Crohns Colitis. 2020;14(8):1162 1171. (doi: 10.1093/ecco-jcc/jjaa036) 32103246

[9] Kim JJ Simpson N Klipfel N DeBose R Barr N Laine L. Cytomegalovirus infection in patients with active inflammatory bowel disease. Dig Dis Sci. 2010;55(4):1059 1065. (doi: 10.1007/s10620-010-1126-4) 20112061

[10] Kishore J Ghoshal U Ghoshal UC Infection with cytomegalovirus in patients with inflammatory bowel disease: prevalence, clinical significance and outcome. J Med Microbiol. 2004;53(11):1155 1160. (doi: 10.1099/jmm.0.45629-0) 15496396

[11] Chaemsupaphan T Sattayalertyanyong O Limsrivilai J. Diagnostic performance of noninvasive tests for cytomegalovirus ileocolitis: a systematic review and meta-analysis. Intest Res. 2025;23(2):213 224. (doi: 10.5217/ir.2024.00136) 39806773 PMC12081080

[12] Harbord M Eliakim R Bettenworth D Third European evidence-based consensus on diagnosis and management of ulcerative colitis. Part 2: Current management. J Crohns Colitis. 2017;11(7):769 784. (doi: 10.1093/ecco-jcc/jjx009) 28513805

[13] Garrido E Carrera E Manzano R Lopez-Sanroman A. Clinical significance of cytomegalovirus infection in patients with inflammatory bowel disease. World J Gastroenterol. 2013;19(1):17-25. (doi: 10.3748/wjg.v19.i1.17) PMC3545225 23326158

[14] Singh G Rentsch C Beattie W Christensen B Macrae F Segal JP. Long-term follow up of patients treated for inflammatory bowel disease and cytomegalovirus colitis. Diagnostics (Basel). 2024;14(18):2030. (doi: 10.3390/diagnostics14182030) PMC11431378 39335709

[15] Zhang C Krishna SG Hinton A Arsenescu R Levine EJ Conwell DL. Cytomegalovirus-related hospitalization is associated with adverse outcomes and increased health-care resource utilization in inflammatory bowel disease. Clin Transl Gastroenterol. 2016;7(3):e150. (doi: 10.1038/ctg.2016.10) PMC4822090 26963000

[16] Hendler SA Barber GE Okafor PN Chang MS Limsui D Limketkai BN. Cytomegalovirus infection is associated with worse outcomes in inflammatory bowel disease hospitalizations nationwide. Int J Colorectal Dis. 2020;35(5):897 903. (doi: 10.1007/s00384-020-03536-8) 32124046

[17] Varani S Landini MP. Cytomegalovirus-induced immunopathology and its clinical consequences. Herpesviridae. 2011;2(1):6. (doi: 10.1186/2042-4280-2-6) 21473750 PMC3082217

